# Editorial: One health care in psychiatric and neurological diseases

**DOI:** 10.3389/fpsyt.2024.1399709

**Published:** 2024-03-20

**Authors:** Júlio Belo Fernandes, Cristina Baixinho, Tiago F. Outeiro, Catarina Godinho

**Affiliations:** ^1^ Nurs Lab, Almada, Portugal; ^2^ Egas Moniz Center for Interdisciplinary Research (CiiEm), Egas Moniz School of Health & Science, Almada, Portugal; ^3^ Nursing School of Lisbon, Lisbon, Portugal; ^4^ Nursing Research, Innovation and Development Centre of Lisbon (CIDNUR), Lisbon, Portugal; ^5^ Department of Experimental Neurodegeneration, Center for Biostructural Imaging of Neurodegeneration, University Medical Center Göttingen, Göttingen, Germany; ^6^ Max Planck Institute for Multidisciplinary Sciences, Göttingen, Germany; ^7^ Translational and Clinical Research Institute, Faculty of Medical Sciences, Newcastle University, Newcastle upon Tyne, United Kingdom; ^8^ Scientific Employee With an Honorary Contract at Deutsches Zentrum für Neurodegenerative Erkrankungen (DZNE), Göttingen, Germany

**Keywords:** one health, one medicine, mental disorders, neurological diseases, environmental health

## 1 Introduction

In healthcare, the convergence of medical disciplines to address complex conditions has become increasingly paramount. The paradigm of One Health Care, an integrated approach encompassing human, animal, and environmental health (1, 2), has gained significant traction. While traditionally applied to infectious diseases and zoonoses, its principles are now extending to encompass psychiatric and neurological disorders. This editorial explores the significance of adopting a One Health approach in managing psychiatric and neurological diseases, elucidating its potential benefits and challenges.

Psychiatric and neurological disorders represent a significant burden on global health (3, 4). Conditions such as depression, schizophrenia, Alzheimer’s disease, Parkinson’s disease, and stroke not only affect individuals but also have profound impacts on families, communities, and healthcare systems worldwide. Despite advancements in medical sciences, these disorders often present multifaceted challenges, requiring comprehensive strategies beyond conventional medical interventions.

The principles of One Health advocate for a holistic understanding of health, emphasizing the interconnectedness of humans, animals, and the environment. This approach acknowledges the intricate interplay between biological, psychological, and environmental factors influencing disease onset, progression, and management in psychiatric and neurological diseases. By embracing a multidisciplinary perspective, One Health endeavors to bridge silos across medical specialties, fostering collaboration among healthcare professionals, scientists, policymakers, and other stakeholders.

In addition to the imperative of embracing One Health principles in psychiatric and neurological care, there exists a pressing need for innovation in treatment modalities (5, 6). Developing interventions that are not only effective but also engaging is crucial to promoting therapeutic engagement and optimizing patient outcomes. Within the realm of psychiatric and neurological disorders, the pursuit of novel approaches to care is paramount to addressing the diverse needs and preferences of individuals undergoing treatment.

The *One Health Care in Psychiatric and Neurological Diseases* Research Topic presented a suitable platform for exploring innovative interventions. Among the published contributions is a protocol to assess the feasibility of adapted Portuguese folk dance as a post-stroke rehabilitative activity (Fernandes et al.), which stands out as a pioneering initiative in neurological rehabilitation. By adapting traditional folk dance as a rehabilitative activity, the protocol seeks to harness the therapeutic potential of cultural traditions to promote motor recovery, cognitive function, and psychosocial well-being among stroke survivors.

Several other studies shed light into essential facets of psychiatric and neurological care. The narrative review investigating disparities in neurological contributors to the national burden of disease underscores the need for targeted interventions and public health initiatives to address conditions such as migraine headaches, cerebrovascular disease, and dementia (Almubaslat et al.). By elucidating the epidemiological landscape and disparities in access to care, the review provides valuable insights for policymakers, healthcare professionals, and researchers striving to mitigate the impact of neurological disorders on public health.

Furthermore, studies examining the role of complementary and alternative medicine in managing psychiatric disorders offer novel perspectives on integrative approaches to mental health care. The meta-analysis exploring the effects of neurobehavioral support programs on mental health outcomes (Menhas et al.) highlights the potential benefits of non-pharmacological interventions in addressing psychological distress and enhancing well-being. Similarly, scientometric analyses (Zhao et al.) and systematic reviews (Zhao et al.) examining complementary and alternative medicine therapies for depression and anxiety provide comprehensive insights into emerging trends, research priorities, and evidence-based recommendations for holistic treatment approaches.

The inclusion of mind-body exercises in psychiatric and neurological care represents another promising avenue for promoting mental health and emotional well-being. The systematic review and network meta-analysis (Dong et al.) evaluating the effects of mind-body exercise on anxiety and depression in older adults underscore the therapeutic potential of practices such as yoga, tai chi, and qigong in alleviating symptoms. By synthesizing evidence from diverse studies, the meta-analysis informs clinical decision-making and underscores the importance of personalized, multimodal interventions in mental health care.

The diverse studies in the Research Topic exemplify the spirit of innovation, collaboration, and interdisciplinary inquiry inherent to One Health’s psychiatric and neurological care approaches. Adopting One Health principles represents a paradigm shift towards more inclusive, holistic approaches prioritizing interconnectedness, collaboration, and compassion. By embracing the complexity of health through interdisciplinary dialogue and partnership, stakeholders can collectively address the multifaceted challenges of psychiatric and neurological diseases, fostering resilience, equity, and well-being across species and ecosystems. As we navigate the intricacies of mental health and neurobiology, let us heed the call of One Health to unite in our pursuit of a healthier, more compassionate world for all.

By fostering dialogue, disseminating knowledge, and advancing evidence-based practice, researchers, clinicians, and policymakers can collectively address the complex challenges of psychiatric and neurological disorders, promoting resilience, equity, and well-being across diverse populations. As we navigate the evolving landscape of mental health and neurobiology, let us embrace innovation, cultivate partnerships, and advocate for compassionate, patient-centered care that honors every individual’s inherent dignity and worth.

## Author contributions

JF: Writing – original draft, Writing – review & editing. CB: Writing – original draft, Writing – review & editing. TO: Writing – original draft, Writing – review & editing. CG: Writing – original draft, Writing – review & editing.
